# Early Pregnancy Screening for Women at High-Risk of GDM Results in Reduced Neonatal Morbidity and Similar Maternal Outcomes to Routine Screening

**DOI:** 10.1155/2020/9083264

**Published:** 2020-01-29

**Authors:** Erin Clarke, Thomas J. Cade, Shaun Brennecke

**Affiliations:** ^1^Pregnancy Research Centre, Department of Maternal-Fetal Medicine, Royal Women's Hospital, Melbourne, VIC, Australia; ^2^Department of Obstetrics and Gynaecology, University of Melbourne, Melbourne, VIC, Australia

## Abstract

The Australasian Diabetes in Pregnancy Society recommends screening high-risk women for gestational diabetes mellitus (GDM) before 24 weeks gestation, under the assumption that an earlier diagnosis and opportunity to achieve normoglycemia will minimize adverse outcomes. However, little evidence exists for this recommendation. The study objective was to compare the pregnancy outcomes of high-risk women diagnosed with GDM before 24 weeks gestation and routinely diagnosed women after 24 weeks gestation. A retrospective audit was conducted of all pregnancies diagnosed with GDM using International Association of Diabetes and Pregnancy Study Groups criteria over 12 months at a tertiary Australian hospital. Adverse perinatal outcomes were compared between “Early GDM” diagnosed before 24 weeks (*n* = 133) and “Late GDM” diagnosed from 24 weeks (*n* = 636). Early GDM had a significantly lower newborn composite outcome frequency (hypoglycemia, birth trauma, NICU/SCN admission, stillbirth, neonatal death, respiratory distress, and phototherapy) compared to Late GDM (20.3% vs. 30.0%, *p* = 0.02). Primary cesarean, hypertensive disorders, postpartum hemorrhage, birthweight >90th percentile, macrosomia, and preterm birth frequencies were not significantly different between groups. Therefore, high-risk women diagnosed with GDM in early pregnancy were not more likely to have an adverse outcome compared to routinely diagnosed women. As they are a high-risk group, this may indicate a possible benefit to the early diagnosis of GDM.

## 1. Introduction

Gestational diabetes mellitus (GDM) is defined as impaired glucose tolerance less than overt diabetes first detected during pregnancy [1]. Adverse outcomes include macrosomia, preterm birth, cesarean section and preeclampsia [2]. GDM affects up to 25% of pregnancies worldwide [3], and 13% in Australia [4]. This number is increasing in Australia, reflecting trends of increasing maternal age, high BMI, and recent changes in screening guidelines [4–7].

In Australia, it is recommended to conduct universal screening for GDM between 24–28 weeks gestation, using a 75 g 2 hour oral glucose tolerance test (OGTT) [8]. High-risk women (defined by Australasian Diabetes in Pregnancy Society (ADIPS) risk factors, such as previous GDM, BMI >35 kg/m^2^, or age ≥40 years) are recommended to receive early screening before 24 weeks [8]. The goal of this early screening is to detect undiagnosed diabetes or GDM at an earlier stage of pregnancy, under the assumption that an earlier opportunity for achieving normoglycemia will minimise adverse outcomes.

There is a paucity of evidence examining the outcomes of high-risk women who are screened early and test positive for GDM. The International Association of the Diabetes and Pregnancy Study Groups (IADPSG) formulated current diagnostic criteria to standardise GDM screening globally, based on associations between hyperglycemia and adverse outcomes in women >24 weeks gestation [2]. These criteria are used for early screening despite a lack of evidence to support this [9]. Our hospital is well positioned to address this research gap as we have adopted the new IADPSG criteria. Therefore, patient groups are not confounded with varying definitions of GDM and there is no clinician bias as to whom to screen, both of which are common confounders in previous studies in other settings.

We aimed to examine the characteristics and adverse pregnancy outcomes of high-risk women screened and diagnosed with GDM before 24 weeks gestation compared to women screened and diagnosed after 24 weeks gestation, using current IADPSG diagnostic criteria.

## 2. Materials and Methods

A retrospective cohort study was performed on women diagnosed with GDM who delivered over a 12-month period in 2016 at a tertiary maternity hospital in Melbourne, Australia. A 75 g, 2 hour OGTT diagnosed GDM using IADPSG criteria, as per the ADIPS guidelines, if at least one of; fasting glucose 5.1–6.9 mmol/L, 1 hour glucose ≥10 mmol/L, and/or 2 hour glucose 8.5–11 mmol/L. Having one “high” risk or two “moderate” risk factors warranted early screening before 24 weeks gestation, with exact timing at clinician discretion. ADIPS risk factors were determined and recorded at antenatal booking visit. These included: previous GDM, age ≥40 years, BMI >35 kg/m^2^ (height and weight measured at booking visit) first degree family history of diabetes mellitus, previous macrosomia, polycystic ovarian syndrome, corticosteroid or antipsychotic use, and nonCaucasian ethnicity [8]. Otherwise, universal screening occurred for all undiagnosed women between 24 and 28 weeks gestation.

Women with multiple pregnancies and incomplete documentation were excluded. Additionally, overt diabetes in pregnancy was considered a separate condition to GDM and these women were excluded. The 2006 World Health Organisation criteria were used to diagnose diabetes in pregnancy if the fasting glucose was >6.9 mmol/L and/or 2 hour glucose was >11 mmol/L on the 75 g OGTT [1].

After diagnosis, women attended a three-hour group education session run by a diabetes educator, dietician and physiotherapist. Self-monitoring of blood glucose, nutrition, and exercise were discussed. Fortnightly review by an obstetrician experienced in GDM management occurred, with endocrinologist input if diagnosis occurred before 18 weeks gestation. The diabetes educator phoned women on nonclinic weeks to assess glycemic control. Women were offered individual dietician follow-up.

Desired blood glucose levels (BGLs) were <5.0 mmol/L and < 6.7 mmol/L for fasting and two hours postprandial, respectively. Insulin was commenced if dietary and lifestyle changes failed to achieve these targets on three matched occasions within the one week. A starting dose of 4–8 units of insulin isophane at bedtime was prescribed if the fasting BGL was elevated. 4–6 units of insulin aspart was prescribed before meals if the postprandial BGL was elevated. Doses were uptitrated to achieve target BGLs, as directed by an obstetrician.

All women received a growth ultrasound at 32 weeks, and again at 36 weeks if macrosomia was suspected. Induction of labor (IOL) was recommended from 38 weeks for women using insulin with one additional risk factor (suboptimal glycemic control, macrosomia, growth restriction, or hypertension), and from 39 weeks for those using insulin but without risk factors. Otherwise, IOL was recommended at approximately 40 weeks for remaining women with GDM who had not yet delivered.

Subjects were grouped into “Early GDM” (high-risk women diagnosed before 24 weeks gestation) and “Late GDM” (women diagnosed from 24 weeks gestation). Demographic and clinical data were collected prospectively at antenatal appointments and delivery, and retrieved retrospectively from electronic databases and medical records.

Adverse maternal outcomes studied included preeclampsia (new onset blood pressure >140/90 mmHg after 20 weeks gestation, with co-existence of >300 mg/day proteinuria or signs of organ dysfunction), cesarean delivery, induction of labor, third degree tear, postpartum hemorrhage (blood loss of >500 mL) and insulin use.

Adverse neonatal outcomes studied included macrosomia (birthweight >4000 g), large for gestational age (LGA; birthweight >90th percentile adjusted for gestational age and gender for an Australian population), small for gestational age (SGA; birthweight <10th percentile adjusted for gestational age and gender for an Australian population), stillbirth (fetal death in utero after 20 weeks gestation and diagnosis of GDM), neonatal death (death during the postnatal period of hospitalisation), hypoglycemia (BGL <2.6 mmol/L), phototherapy, preterm birth (birth before 37 weeks gestation), admission to neonatal intensive care unit (NICU) or special care nursery (SCN), and birth trauma. A neonatal composite outcome was created which included one or more of all neonatal outcomes excluding LGA, macrosomia, and preterm birth, due to the small frequency of these outcomes in the general obstetric population.

Data were analysed using Stata (Version 9.2, StataCorp). Continuous data were reported as means with standard deviations. Parametric means were compared using the Student's *t* test. Nonparametric means were compared using the Mann–Whitney *U* test. Categorical data were reported as exact numbers and percentages, with outcomes compared using Fisher's exact test. Statistical significance was at *p* < 0.05. No apriori power analysis was performed.

Ethical approval was received from the Royal Women's Hospital Human Research and Ethics Committees (Project AQA17/09, on 01/02/17) and the Monash University Human Research Ethics Committee (Project 8276, on 10/02/17).

## 3. Results

Over the 12 month period of the study, 793 women with GDM delivered. The final sample included 769 women after exclusions for overt diabetes in pregnancy according to WHO criteria (16), incomplete documentation (2), and multiple pregnancy (6). The final sample was grouped as Early GDM (*n* = 133) and Late GDM (*n* = 636). 17.3% of women with GDM were diagnosed before 24 weeks gestation, at an average gestation of 17 weeks (±5.2 weeks).

Baseline characteristics of women included in the study are shown in Table 1. Compared to Late GDM, Early GDM had a higher BMI (29.8 ± 7.3 vs. 26.1 ± 6.3, *p* < 0.0001), were multiparous (62.4% vs. 46.4%, *p* = 0.0008) and had higher insulin use (65.4% vs. 41.2%, *p* < 0.0001). Groups did not differ significantly with regards to maternal age and country of origin.

Adverse maternal outcomes are summarised in Table 2. Early GDM had a significantly higher rate of cesarean section (45.8% vs. 35.5%, *p* = 0.02) than Late GDM, however, significance was lost when isolating primary cesarean (19.5% vs. 19.6%, *p* = 0.08). Instrumental delivery was more frequent in Late GDM (11.3% vs. 19.0%, *p* = 0.03). Early and Late GDM had no significant difference in rates of hypertensive disorders, postpartum hemorrhage, induction of labor and third degree tear.

Neonatal outcomes are listed in Table 2. Early GDM had a significantly lower rate of newborn composite outcome compared to Late GDM (20.3% vs. 30.0%, *p* = 0.02). There were no significant differences in the remaining newborn outcomes of macrosomia, LGA, SGA, and preterm birth. There were no occurrences of birth trauma or neonatal death in Early GDM.

## 4. Discussion

Although treatment of GDM diagnosed after 24 weeks reduces the risk of adverse outcomes [10, 11], there is uncertainty regarding the benefits of screening high-risk women in early pregnancy and which criteria should be used. Current literature suggests these women experience more adverse outcomes than routinely diagnosed women despite treatment [12], albeit with varying diagnostic criteria, heterogenous patient groups and treatment regimens. In contrast, we report that these high-risk women diagnosed in early pregnancy did not have worse outcomes.

Early diagnosis was not associated with higher frequencies of LGA, macrosomia, preterm birth, postpartum hemorrhage, preeclampsia, primary or emergency cesarean and induction of labor. In GDM, these adverse maternal outcomes often occur as a consequence of macrosomia [5]. It is possible that early diagnosis with IADPSG criteria allowed for a longer time frame to achieve normoglycemia via multidisciplinary care and insulin as required, thereby reducing the frequency of macrosomia to that found in Late GDM.

However, Early GDM did have a higher frequency of secondary cesarean delivery, which carries associated morbidity for women. Both groups had no significant difference in common indications for cesarean such as macrosomia, preeclampsia, or IOL. We do not have data for other indications such as cephalopelvic disproportion or abnormalities with placentation. Therefore, this may be due to the significantly higher proportion of multiparous pregnancies seen in Early GDM whereby events in a previous pregnancy could influence delivery method decisions.

Several studies have examined these adverse outcomes of high-risk women [12–17]. A recent Australian study concluded that despite early diagnosis, high-risk women suffered higher rates of preterm delivery, cesarean delivery, preeclampsia, and macrosomia, even after excluding diabetes in pregnancy [12]. On the contrary, studies which used different diagnostic criteria have found that early diagnosed GDM was associated with a similar frequency of adverse outcomes, such as macrosomia, to routinely diagnosed GDM [3, 14] and even women without GDM [18]. These studies did not use current IADPSG criteria, limiting applicability to current practice. A small Australian pilot study using IADPSG criteria has recently highlighted that early diagnosis may be associated with a reduced rate of large for gestational age babies [19] which supports our suggestion that early diagnosis of GDM may be beneficial.

Additionally, Early GDM had a significantly lower frequency of the newborn composite outcome than Late GDM (20.3% vs. 30.0%, *p* = 0.02). This composite included the less common known complications of GDM including neonatal death, stillbirth, birth trauma, hypoglycemia, NICU or SCN admission, respiratory distress and phototherapy. In contrast, a recent Australian study found no difference in the neonatal composite outcome (Apgar <7 at 5 minutes, NICU/SCN admission, neonatal hypoglycemia or major birth defect) between early and routinely diagnosed GDM groups [20]. Our Early GDM group had a 4.6% lower frequency of NICU/SCN admission and a 7% lower frequency of neonatal hypoglycemia compared to theirs [20], which may contribute to the difference in the neonatal composite outcome frequency. These results may relate to how our study also had a 11.2% lower frequency of birthweight >90th percentile, which is a known risk factor for hypoglycemia and admission to the NICU/SCN [5]. However, their study sample is a heterogenous group over 10 years comprised of several changes in the GDM diagnostic criteria and likely different management strategies, such as criteria for admission to the NICU/SCN.

Alternatively, it is possible that this reduction in neonatal morbidity is a result of an opportunity to achieve normoglycemia earlier in pregnancy, an opportunity created by the adoption of early screening with IADPSG criteria. This finding may be due to chance as these are less common outcomes, and warrants further investigation on a larger scale. Nevertheless, both studies support the conclusion that high risk women with an early diagnosis of GDM did not experience worse adverse outcomes than routinely diagnosed GDM [20]. As these women are high risk, this suggests a possible benefit to early screening.

The Early GDM group were diagnosed at an average 17 weeks gestation. This may represent a beneficial screening time point for high-risk women, affording intervention prior to the development of a functional fetal endocrine pancreas and subsequent macrosomia [21]. We suggest that clinical practice should continue to follow the ADIPS recommendations of screening at first opportunity for high-risk women [8] until further research establishes the benefits of specific time points.

In this study, Early GDM required a higher use of insulin compared to Late GDM, as commonly reported elsewhere [12–14, 17, 22]. This may be because early diagnosis affords a longer period to commence insulin, or because high-risk women are characterised by an insulin-resistant phenotype due to factors such as high BMI [23]. As first trimester fasting dysglycemia is independently associated with adverse outcomes [24], it is plausible that the frequency of adverse outcomes could have been higher in the absence of early diagnosis with IADPSG criteria. There is also concern about possible “over-treatment” that may restrict fetal growth [3, 19, 25]. Reassuringly, our study found no difference in SGA between groups.

Early diagnoses comprised 17.3% of all GDM diagnoses in our study. This is lower than the 27.4% recently reported in Australia [12] and internationally [13, 15, 16, 26]. It is difficult to assess the true prevalence between studies with different diagnostic criteria and populations. Regardless, Early GDM constitutes a significant proportion of GDM diagnoses and emphasises the need for rigorous evidence to justify early screening. It is an opportunity to potentially improve outcomes for many women, however, may result in higher costs due to increased antenatal visits, insulin use [27], growth scans and secondary cesarean deliveries. Therefore, it must be justified from an economic perspective.

Additionally, early screening must also be justified with consideration of women's experiences. Early GDM had no difference in adverse maternal outcomes and so satisfaction of the early screening and management period could be influential in deciding for early or routine screening.

Advantages of our study are that it used IADPSG criteria and has no screening bias. All women were screened for GDM, with the same screening and diagnostic criteria. Many studies have heterogeneous groups comprised of varying screening and diagnostic practices, meaning assessment of early intervention is difficult. Many studies are also confounded by including overt diabetes in pregnancy, which has different adverse outcomes to GDM [12]. We excluded these women and so outcomes are reflective of GDM.

This study's limitations include the retrospective nature and inability to assess adherence to treatment and glycemic control. Poor glycemic control may contribute to adverse outcomes, given that hyperglycemia is independently associated with morbidity [2]. Additionally, the study did not have adequate power to detect differences in rare outcomes such as stillbirth. Early GDM had a higher frequency of stillbirth, and so these results may hint a true association between Early GDM and stillbirth, despite early treatment. We could not quantify the magnitude of adverse outcome improvement in Early GDM without a control group for comparison.

## 5. Conclusions

This study examined the outcomes of high-risk women screened and diagnosed with GDM in early pregnancy with current IADPSG diagnostic criteria. Our study found that these women did not experience more adverse outcomes than women diagnosed ≥24 weeks, suggesting a possible benefit to early screening. Therefore, we support the continued use of the ADIPS guidelines which recommend screening at first opportunity for high-risk women. A future randomized control trial examining the benefit of early diagnosis is a key research priority, especially with rising rates of advanced maternal age, high BMI, and GDM diagnoses. We eagerly await the results of an upcoming study in the near future to guide clinical practice [19, 25]. This clinical justification must also be supported by an economic analysis and patient experience to justify the burden of early screening.

## Figures and Tables

**Table 1:** Characteristics of the maternal and newborn study participants. Early GDM had a higher BMI, more multiparous pregnancies, higher use of insulin, and a lower gestational age.

Characteristic	Early GDM	Late GDM	Significance
	Mean ± SD *n* (%)	Mean ± SD *n* (%)	
Number of women	133 (17.3)	636 (82.7)	

Age (years)	32.9 ± 4.9	32.2 ± 4.7	0.09

Body mass index (m/kg^2^)	29.8 ± 7.3	26.1 ± 6.3	0.0001

Country of birth			
Australia	36 (27.1)	187 (29.4)	
India	24 (18.1)	85 (13.3)	
Pakistan	10 (7.5)	27 (4.2)	
Bangladesh	6 (4.5)	13 (2.0)	
China	6 (4.5)	38 (5.9)	
Vietnam	3 (3.5)	40 (6.3)	
Other	48 (34.8)	246 (38.9)	

First degree family history of diabetes	70 (52.6)	^∗^	

Polycystic ovarian syndrome	22 (16.5)	^∗^	

Previous GDM	51 (38.3)	^∗^	

Early pregnancy OGTT values (mmol/L)			
Fasting glucose	4.9 ± 0.6	^∗^	
1 hour glucose	10.2 ± 1.7		
2 hour glucose	8.1 ± 1.6		

HbA1c (%)	5.2 ± 0.4	^∗^	

Parity			
Primiparous	50 (37.6)	341 (53.6)	
Multiparous	83 (62.4)	295 (46.4)	0.0008

GDM^†^ management			
Diet only	46 (34.6)	374 (58.8)	
Insulin	87 (65.4)	262 (41.2)	<0.0001

Newborn gestational age at delivery (weeks)	37.8 ± 2.1	37.9 ± 2.2	0.03
Newborn birth weight (g)	3164.9 ± 610.1	3190.0 ± 595.6	0.40

^†^GDM: Gestational diabetes mellitus. ^∗^Data not available.

**Table 2:** Adverse pregnancy outcomes in early GDM and late GDM. Early GDM had a significant decrease in frequency of newborn composite outcome compared to Late GDM. Composite includes one or more of hypoglycemia, birth trauma, NICU/SCN admission, stillbirth, neonatal death, phototherapy and respiratory distress.

Adverse outcome	Early GDM *n* = 133	Late GDM *n* = 636	Significance
	*n* (%)	*n* (%)	
Cesarean delivery			
Total cesareans	61 (45.8)	226 (35.5)	0.02
Primary cesarean	26 (19.5)	125 (19.6)	0.08
Emergency cesarean	26 (19.5)	96 (15.1)	0.98

Induction of labor	64 (48.1)	309 (48.6)	0.90

Hypertensive disorders			
Gestational hypertension	5 (3.8)	9 (1.4)	0.08
Pre-eclampsia	3 (2.2)	15 (2.4)	0.60
Total	8 (6.0)	24 (3.8)	0.20

Postpartum hemorrhage	36 (27.1)	171 (26.9)	0.7

Third degree tear	2 (1.5)	18 (2.8)	0.50

Birth weight > 90^th^ percentile	15 (11.3)	57 (9.0)	0.40

Birth weight < 10^th^ percentile	12 (9.0)	55 (8.7)	0.80

Macrosomia	7 (5.2)	35 (5.5)	0.90

Hypoglycemia	7 (5.3)	49 (7.7)	0.30

Preterm birth < 37 weeks	14 (10.5)	89 (13.9)	0.30

Birth trauma	0 (0)	3 (0.5)	
Instrumental delivery	15 (11.3)	121 (19.0)	0.03

NICU^†^ admission	5 (3.8)	47 (7.4)	0.10
SCN^‡^ admission	13 (9.8)	60 (9.4)	0.87
NICU or SCN admission	18 (13.6)	107 (16.8)	0.40

Stillbirth	3 (2.3)	4 (0.6)	0.10

Respiratory distress	2 (1.5)	11 (1.7)	0.80

Phototherapy	1 (0.8)	16 (2.5)	0.20

Neonatal death	0 (0)	1 (0.2)	

Newborn composite outcome	27 (20.3)	191 (30.0)	0.02

^†^NICU: Neonatal intensive care unit. ^‡^SCN: Special care nursery.

## Data Availability

The data used to support the findings of this study are available from the corresponding author upon request.
